# Cloning and functional characterization of the legumin A gene (*EuLEGA*) from *Eucommia ulmoides* Oliver

**DOI:** 10.1038/s41598-024-65020-5

**Published:** 2024-06-19

**Authors:** Lina Zheng, De-Gang Zhao

**Affiliations:** 1 The Key Laboratory of Plant Resource Conservation and Germplasm Innovation in Mountainous Region (Ministry of Education), College of Life Sciences/Institute of Agro-Bioengineering, Guizhou University, Guiyang, 550025 Guizhou Province China; 2 Guizhou Plant Conservation Technology Center, Biotechnology Institute of Guizhou, Guizhou Academy of Agricultural Sciences, Guiyang, 550006 Guizhou Province China

**Keywords:** *Eucommia ulmoides*, Legumin A, Legumin A-encoding gene, Transgene, Biochemistry, Biological techniques, Biotechnology, Cell biology, Chemical biology, Molecular biology, Plant sciences

## Abstract

Legumin A is a seed storage protein that provides nutrients for seed germination. The purpose of this study was to describe the structure and expression pattern of the *EuLEGA* gene in *Eucommia ulmoides* Oliver (*E. ulmoides*) and to infer its functional role. The 1287 bp coding sequence of the *EuLEGA* CDS of the *EuLEGA* gene, encoding a protein containing 428 amino acid residues, was cloned. The structure predicted that the protein belonged to the RmlC (deoxythymidine diphosphates, dTDP)-4-dehydrorhamnose 3,5-epimerase)-like cupin conserved domain family, which contains both RmlC, a key enzyme for the synthesis of rhamnose and legumin A. The overexpression (OE) vector of the *EuLEGA* gene was constructed and genetically transformed into tobacco and *E. ulmoides*; the RNA interference (RNAi) vector of the *EuLEGA* gene was constructed and genetically transformed into *E. ulmoides*; and the contents of legumin A and rhamnose were detected. The results showed that the *EuLEGA* gene could significantly increase the content of legumin A in transgenic tobacco leaves and transgenic *E. ulmoides* regenerative buds, and the OE of this gene in *E. ulmoides* could promote an increase in rhamnose content. RNAi caused a significant decrease in the legumin A content in the regenerated buds of *E. ulmoides*. These was a significant increase in legumin A in the transgenic tobacco seeds, and these results indicate that the expression of the *EuLEGA* gene is closely related to the accumulation of legumin A. Subcellular localization studies revealed that EuLEGA is localized to the cytoplasm with the vacuolar membrane. Analysis of the *EuLEGA* gene expression data revealed that the expression level of the *EuLEGA* gene in the samaras was significantly greater than that in the leaves and stems. In addition, the study also demonstrated that GA_3_ can upregulate the expression levels of the *EuLEGA* gene, while ABA and MeJA can downregulate its expression levels.

## Introduction

A unique ancient tree species in China, *Eucommia ulmoides* Oliver (*E. ulmoides*), has multiple functions^1–3^. First, *E. ulmoides* is another important rubber-producing plant in addition to the rubber tree *Hevea brasiliensis* (*H. brasiliensis*). The molecular structure of *E. ulmoides* rubber is trans-1,4-polyisoprene, an isomer of the homeopathic 1,4-polyisoprene of *H. brasiliensis* rubber^4–7^. *E. ulmoides* rubber has been widely used in many fields such as the environment, agriculture and medicine; its unique thermal stability, excellent mechanical properties and biodegradability make it a new type of material that plays an important role in sound insulation, tires and composite films^8^. Second, *E. ulmoides* has many pharmacological functions. The primary bioactive constituents of *E. ulmoides* include triterpenoids and flavonoids, among others. These key components exert therapeutic effects on osteoporosis by modulating bone marrow mesenchymal stem cells and osteoblasts to promote bone formation while concurrently inhibiting osteoclast-mediated bone resorption and adipocyte differentiation^9^. In addition, many advances have been made in immune on *E. ulmoides*, and some studies have shown that *E. ulmoides* can increase immune function and protect liver function in mice and is expected to be applied in medical treatment^10^. Moreover, the use of *E. ulmoides* leaf extract as a dietary supplement has been demonstrated to augment the immune response, disease resistance capacity, and intestinal antioxidant capacity in tilapia (*Oreochromis niloticus*), chickens, and *Scophthalmus maximus*^11–13^. In recent years, *E. ulmoides* has been utilized in health care products such as black tea, green tea, oolong tea and other products, within the Chinese market^14^.

The cupin protein superfamily, named after the conserved beta barrel structure, is a large family with a wide range of functions. The family includes two types of proteins: monocupins and bicupins^15^. These two types of proteins are diverse. For example, monocupins include dioxygenases, auxin binding proteins and nuclear proteins. Bicupins include phosphomannose isomerase, globulin seed storage proteins and seed storage proteins (SSPs)/sucrose-binding protein (SBP)^16,15^. Among these, phosphomannose isomerase is involved in rhamnose synthesis in plants; seed storage globulins and sucrose-binding proteins associated with seed globulins found in seeds belong to the bicupins family^17,18^. Members of the cupin family play a very important role in the development of plant roots, leaves, flowers, fruits, and seeds as well as in the stress response^19–21^. Cupin proteins have received much attention for their use in crop improvement, with some members enhancing plant growth, development and resistance to adversity, thereby increasing crop yields. Due to the expansion of cupin genes during evolution, the roles of cupin family members vary widely among species^22^.

Legumin, a member of the globulin class also known as 11S globulin, is a class of storage protein in many seed plants^23^. Immunoassays performed by Iwabuchi et al.^24^ revealed that 7S and 11S globulins accounted for approximately 50% of the total protein in soybean seeds, with 11S globulin representing 52% of globulin in soybean seeds. The 11S globulin precursor exists as a trimer in the endoplasmic reticulum and is transported to the vesicle before being stored in the vesicle as a hexamer^25^. The vesicle consists mainly of six pairs of subunits of 11S globulin, each pair of subunits linked by alpha and beta subunits via disulphide bonds^26^. Legumin is classified into two subfamilies, A and B, based on whether it is methionine-rich^27^. 11S globulin shares the same conserved structural domains as dTDP-6-deoxy-d-xylo-4-hexulose 3,5-epimerase (RmlC) enzymes and belongs to the cupin superfamily^28,29^. Additionally, legumin is a member of the major family of proteins involved in allergens, including 2S albumin, 7S globulin, and nonspecific lipid transfer protein (nsLTP)^30,31^. Currently, the utilization of 11S globulin in the medical field is extensive, encompassing protein structure modification through binding with other substances to mitigate sensitization associated with 11S globulin^32^. It has previously been shown that there are two major storage proteins in soybean seeds, 7S and 11S, that affect the content and abundance of total proteins; the authors also indicated that the absence of a particular globulin polypeptide (11SA4) in their study resulted in significantly increased abundance of lectin and that the exact biological function of lectin is related to seed germination^33^. Additional studies have shown that the effect of 11S on protein content is particularly important^34^. Consequently, legumin is important for seed growth and is of interest for studying the ability of *E. ulmoides* to survive and improve its adaptation to the environment.

Previously, our group identified the STLNSHNLPILR peptide in rubber particle proteins, determined the gene containing this peptide from the whole-genome database of *E. ulmoides* constructed in our laboratory^35^, and analysed the gene-encoded protein to identify the conserved structural domain of the Cupin_RmlC-like superfamily. We designed primers based on the gene coding sequence, cloned the cDNA coding sequence of the *EuLEGA* gene using RT‒PCR, and investigated the subcellular localization of the gene product, gene expression characteristics, expression regulation and biological functions. The *EuLEGA* gene was overexpressed in tobacco through transgenic technology, and its function was preliminarily analysed through OE and RNAi in *E. ulmoides*.

## Results

### Cloning of *EuLEGA* gene in *E. ulmoides*

In this study, total RNA was extracted from the leaves of *E. ulmoides* seedlings, and the gene containing the conserved structural domain of the Cupin_RmlC-like superfamily (named *EuLEGA*) was identified from the *E. ulmoides* whole-genome annotation database constructed by our team. PCR primers were designed, the coding sequences were amplified using RT‒PCR, and the amplified CDSs were 1287 bp in length, encoding 428 amino acid residues.

### Analysis of the structural domains and properties of the protein encoded by the *EuLEGA* gene

Comparison of the NCBI *E. ulmoides* whole-genome annotation database revealed that the gene has four exons and three introns. Conserved structural domain analysis of EuLEGA revealed that the protein has a conserved structural domain belonging to the Cupin_RmIC-like superfamily, which has conserved structural domains catalysing both l-rhamnose synthase (RmlC) and the subunit of legumin 11S^29^. RmlC is the dTDP-l-rhamnose pathway's third enzyme that catalyses the synthesis of l-rhamnose from deoxythymidine diphosphate (dTDP)^36^, the legumin 11S subunit is a storage protein for seeds^37^. Physicochemical analysis of the EuLEGA protein revealed a molecular weight of 48.04 kDa, a theoretical isoelectric point of 9.52, and a molecular formula of C_2088_H_3323_N_647_O_631_S_14_. The EuLEGA signal peptide was predicted to be 97.62% likely to be a signal peptide using the SignalP 4.1 or SignalP 5.0 Server (https://services.healthtech.dtu.dk/services/SignalP-5.0/); analysis of its transmembrane structural domains indicated that the protein has a transmembrane structural domain.

Secondary structure analysis of the EuLEGA protein sequence using the NovoPro online analysis software (https://www.novoprolabs.com/protein-crystallography-service/) showed that the protein encoded by this gene consists of three secondary structures: α-helix, irregularly coiled structure, and β-folded structure. Further modelling of the tertiary structure of the EuLEGA protein using SWISS-MODEL revealed that the tertiary structure of the protein is a crystal structure similar to that of the almond Pru1 protein^38^, suggesting a similarity to the tertiary structure of legumin: the formation of two trimers from the interaction of monomers and further interaction of trimers to form a hexamer^37^.

**Figure 1:**
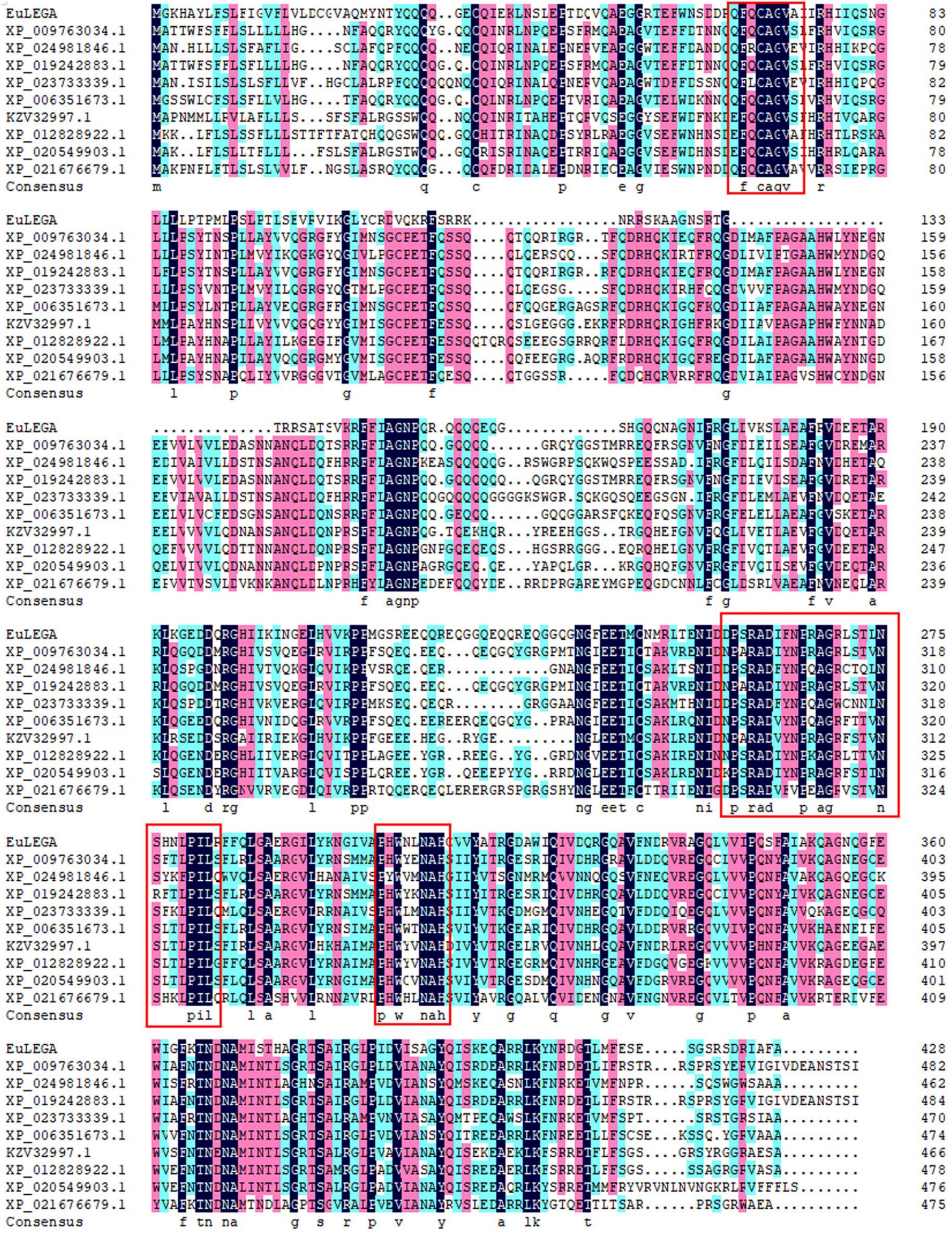
Amino acid comparison of the legumin A protein from *Eucommia ulmoides* with other species of legumin. Note: (> XP_009763034.1 [*Nicotiana sylvestris*]; > XP_024981846.1 [*Cynara cardunculus* var. scolymus]; > XP_019242883.1 [*Nicotiana attenuata*]; > XP_023733339.1 [*Lactuca sativa*]; > XP_006351673.1 [*Solanum tuberosum*]; > KZV32997.1 [*Dorcoceras hygrometricum*]; > XP_012828922.1 [*Erythranthe guttata*]; > XP_020549903.1 [*Sesamum indicum*]; > XP_021676679.1 [*H. brasiliensis*].)

Comparative analysis of the conserved structural domains of the EuLEGA protein with those of legumin from seed plants such as tobacco, artichoke, and rubber tree revealed multiple conserved peptides, as shown in Fig. 1. Conserved peptides (-QFQCAGVA-, -DPSRADIFNPRAGRLS TLNNSHNLPIL-, and -PHWNLNAH-) are highly conserved with those of soy globulins from rice, sunflower, and ginkgo biloba land cotton seed plants^38^.

### Analysis of the transgenic plant material

**Figure 2:**
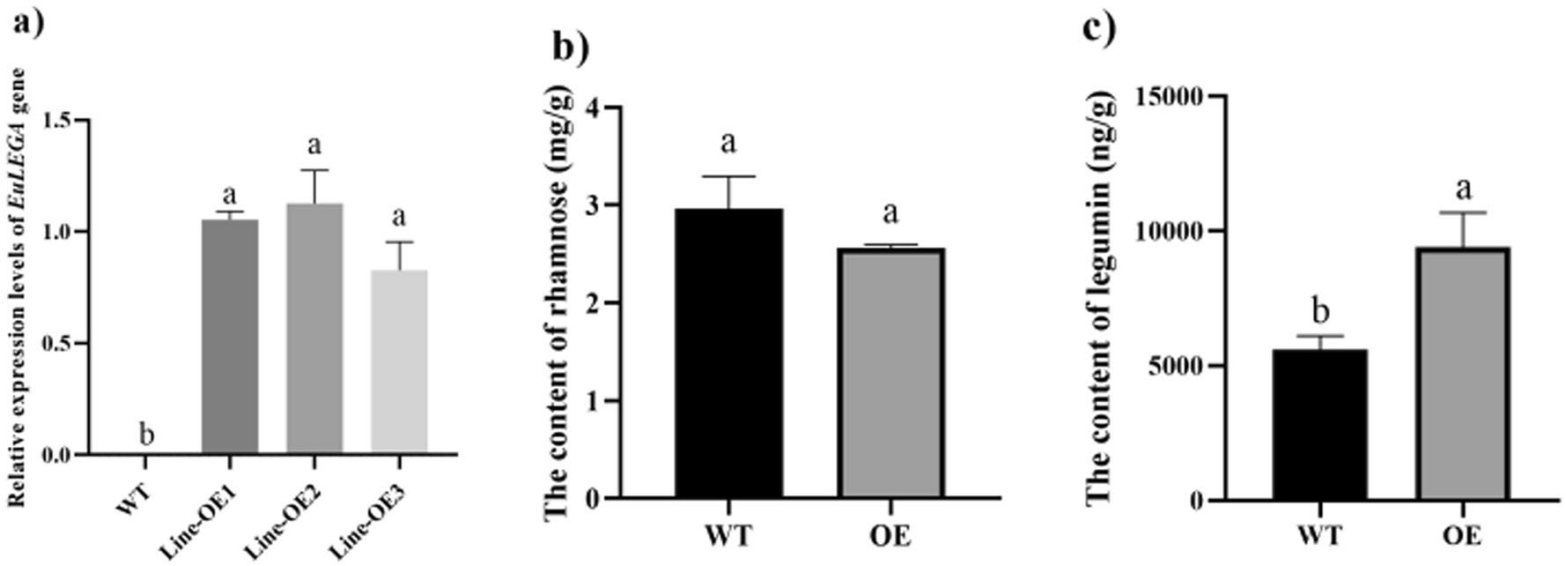
Detection results of related characteristics of tobacco transformed by the legumin A gene from *Eucommia ulmoides*. Note: (**a**) The expression levels of the legumin A gene from *Eucommia ulmoides* in transgenic tobacco leaves; (**b**) The content of rhamnose in transgenic tobacco leaves; (**c**) Legumin A content in transgenic tobacco leaves. Different letters indicate significant differences. *P* < 0.05*.*

After the *EuLEGA-*overexpressing plants were genetically transformed into tobacco, 13 positive plants were obtained via GUS staining and direct PCR verification. Three of these plants were selected for real-time fluorescence-based quantitative PCR to observe their expression levels. The results showed that the expression of this gene significantly increased in tobacco (Fig. 2a), while it was almost nonexpressed in the control wild type (WT). The detection of rhamnose content in overexpressed transgenic tobacco revealed no significant increase (Fig. 2b), suggesting that this gene is not directly related to rhamnose synthesis in tobacco. The detection of the content of legumin 11S revealed a significant increase in comparison with that in the WT (Fig. 2c), suggesting that this gene is closely related to the content of legumin in tobacco.

**Figure 3:**
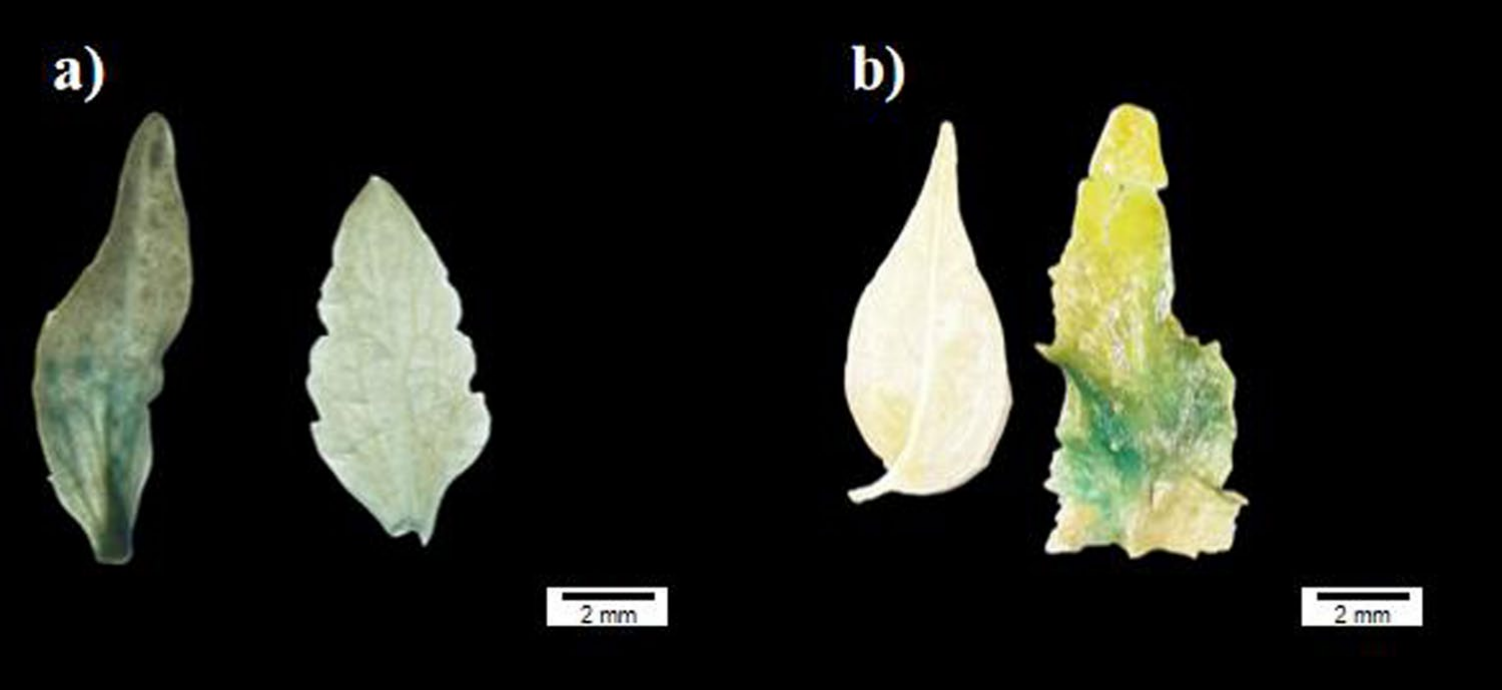
GUS immunostaining of transgenic regenerated buds of *Eucommia ulmoides.* Note: (**a**) GUS validation of regenerated buds of *Eucommia ulmoides* with overexpressing; (**b**) GUS validation of regenerated buds of *Eucommia ulmoides* with RNAi. Scale bar = 2 mm.

**Figure 4:**
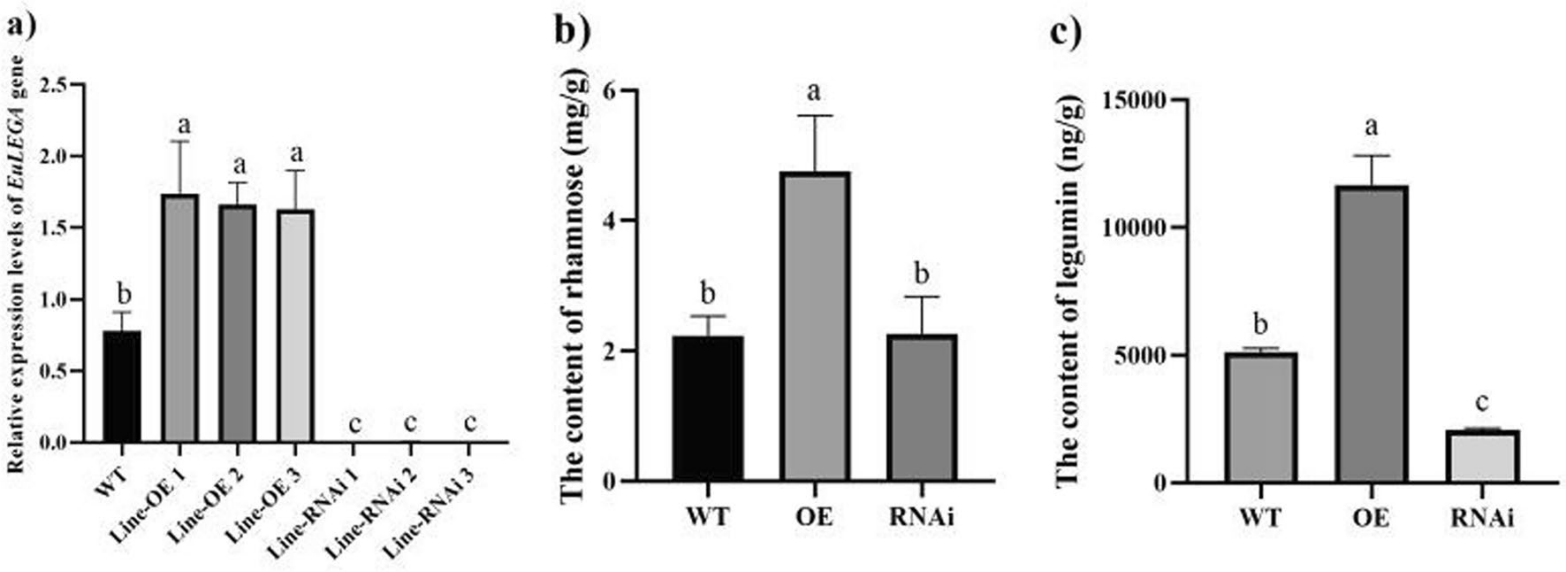
Detection results of related characteristics of the legumin A gene after *Eucommia ulmoides* genetic transformation. Note: (**a**) The expression levels of the legumin A gene from *Eucommia ulmoides* in the regenerated buds of transgenic *Eucommia ulmoides*; (**b**) The content of rhamnose in the regenerated buds of transgenic *Eucommia ulmoides*; (**c**) The content of legumin in transgenic *E. ulmoides* regenerated buds. Different letters indicate significant differences. *P* < 0.05*.*

Subsequently, the OE and RNAi vectors of this gene were genetically transformed into *E. ulmoides* explants, and regenerated buds overexpressing *E. ulmoides* were obtained, which were identified by GUS staining (Fig. 3a). Eleven positive plants were obtained, including 59 regenerated buds from RNAi-treated *E. ulmoides* plants and 6 GUS-stained positive buds (Fig. 3b). *EuLEGA* gene expression was significantly increased in the regenerated buds of transgenic *E. ulmoides* plants and significantly decreased in the regenerated buds of RNAi transgenic *E. ulmoides* plants (Fig. 4a). Analysis of the transgenic *E. ulmoides*-positive plants revealed that the contents of both legumin and rhamnose in the *E. ulmoides*-overexpressing strains increased significantly (Fig. 4b,c), the content of legumin in the RNAi-treated *E. ulmoides* strains decreased significantly (Fig. 4c), and the content of rhamnose did not change significantly (Fig. 4b). This indicates that this gene is not directly related to rhamnose synthesis but rather is associated with legumin synthesis. Interestingly, the presence of *EuLEGA* increased the content of rhamnose.

**Figure 5:**
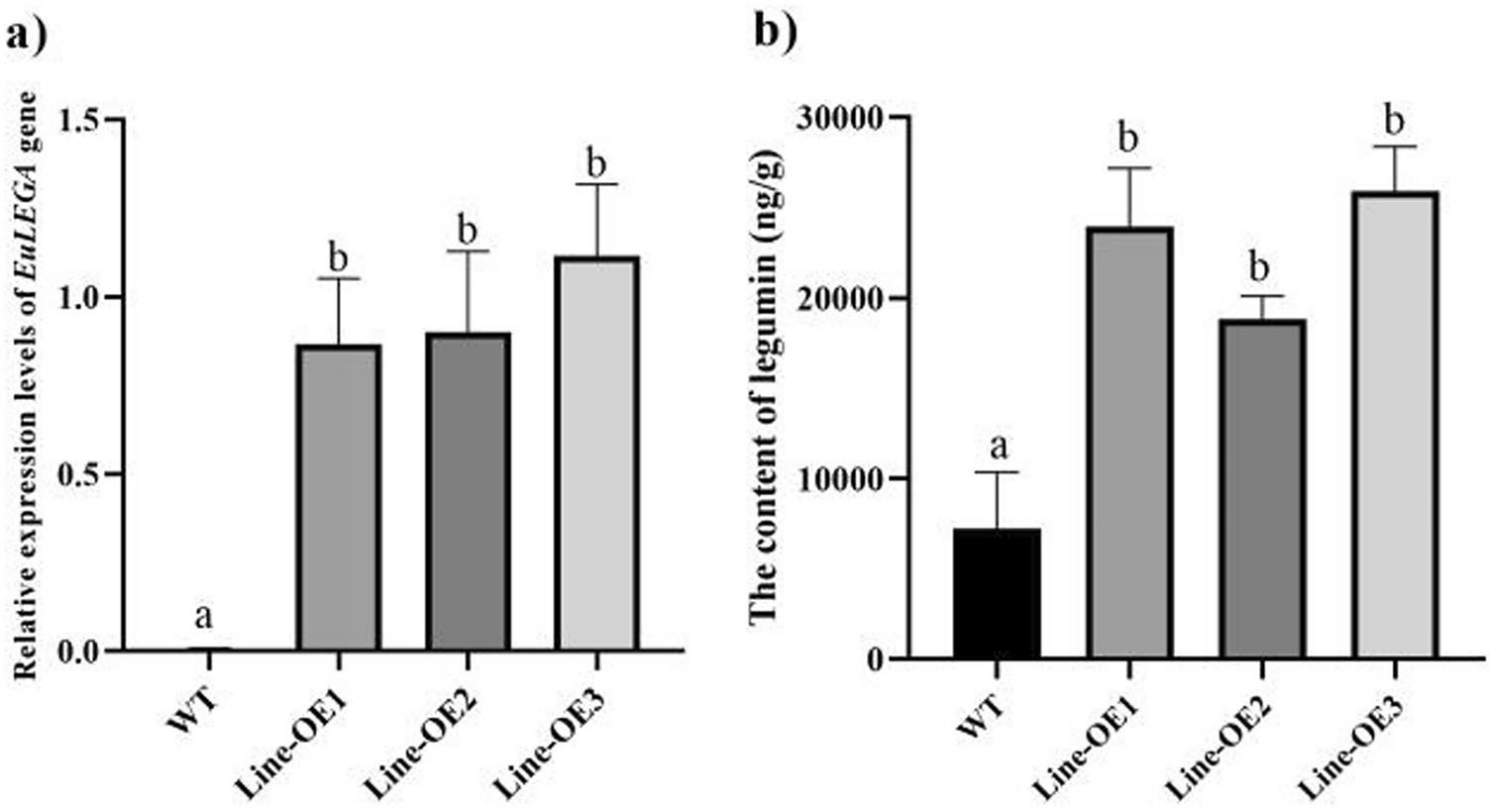
The expression levels of the legumin A gene from *Eucommia ulmoides* and content of legumin in transgenic tobacco seeds. Note: (**a**) The relative expression levels of the legumin A gene from *Eucommia ulmoides*; (**b**) The content of legumin. Different letters indicate significant difference. *P* < 0.05*.*

Transgenic tobacco overexpressing strains were transplanted to soil, allowed to grow, mature and seed, after which the *EuLEGA* gene expression levels and legumin content in the transgenic tobacco seeds were detected (Fig. 5). Compared with that in the WT, the expression of the EuLEGA gene was significantly greater in the Line-OE 3 plants, which increased 194-fold compared with that in the WT. The lowest expression was in the Line-OE 1 plants, which exhibited a 150-fold increase compared with that in the WT and a 167-fold increase in average relative expression compared with that in the WT. The content of legumin also significantly increased compared with that in the type, with the highest content in Line-OE 3 plant (3.59-fold) that of WT, and the lowest content of legumin in the Line-OE 2 plant (2.6-fold) compared with that in the WT plants. The average content of legumin in the transgenic tobacco seeds increased 2.23-fold compared with that in the WT plants. All these results illustrate that the *EuLEGA* gene is associated with the synthesis of legumin in seeds.

### Subcellular localization of proteins encoded by the *EuLEGA* gene

**Figure 6:**
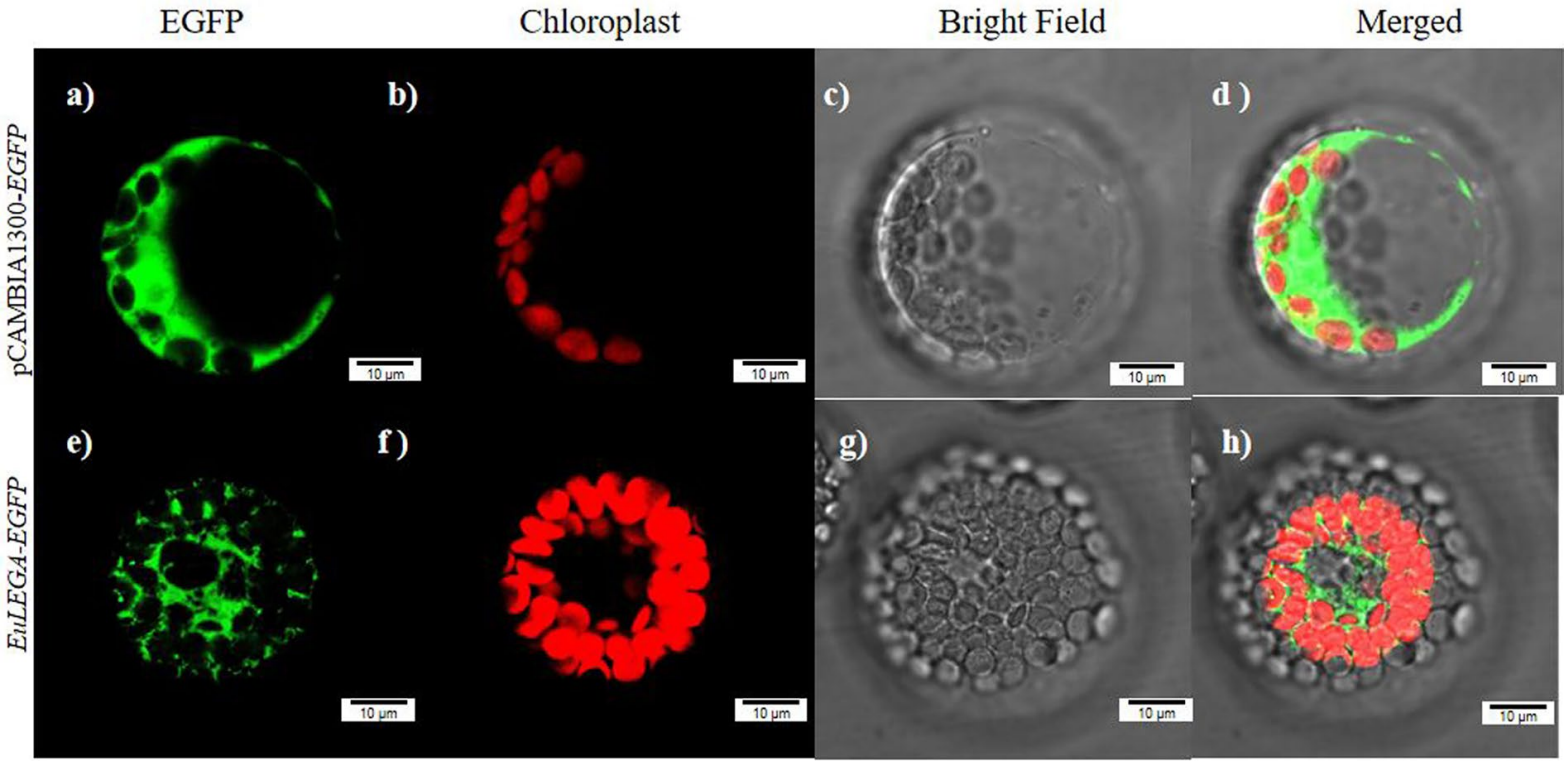
The legumin A protein from *Eucommia ulmoides* subcellular localization. Note: Scale bar = 10 µm.

Subcellular localization of the EuLEGA protein was predicted to be present in vesicles using the Cell-PLoc 2.0 online prediction site. A fusion protein expression vector for the *EuLEGA* and GFP genes was constructed and transformed into *Arabidopsis thaliana* (*A. thaliana*) protoplasts. The pCAMBIA1300 empty vector was used as a control Fig. 6a–d show that the GFP empty load was expressed in the cell membrane and cytoplasm, and from Fig. 6e–h show that the EuLEGA protein was localized in the cytoplasm and vesicle membrane.

### Characterization of *EuLEGA* gene expression

**Figure 7:**
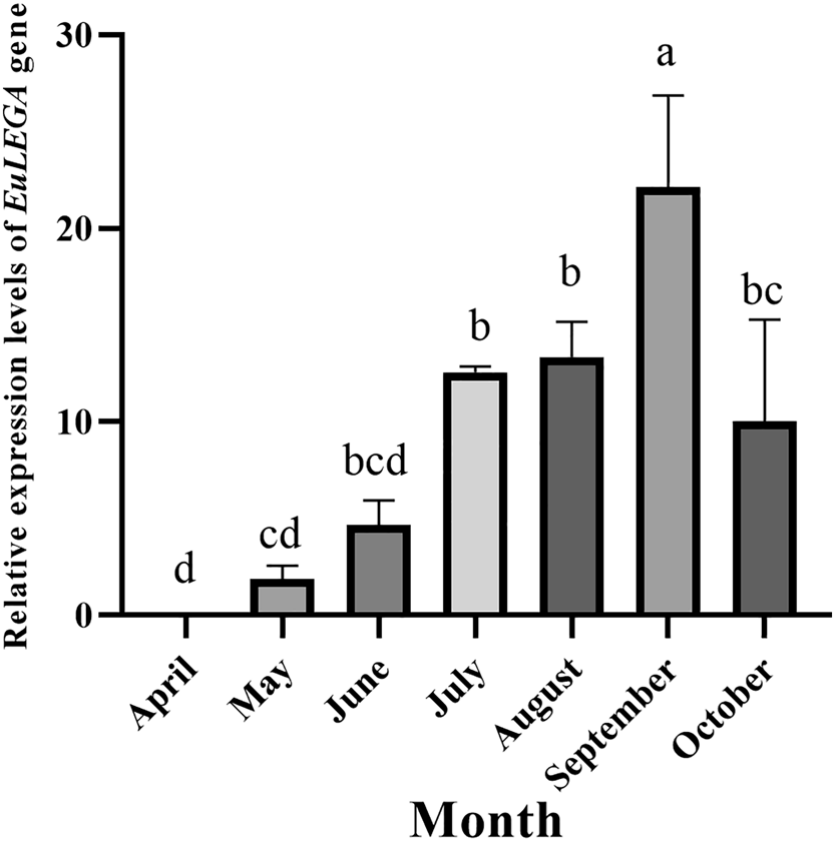
Expression levels of the legumin A gene in *Eucommia ulmoides* samaras from April to October. Note: Different letters indicate significant differences. *P* < 0.05*.*

**Figure 8:**
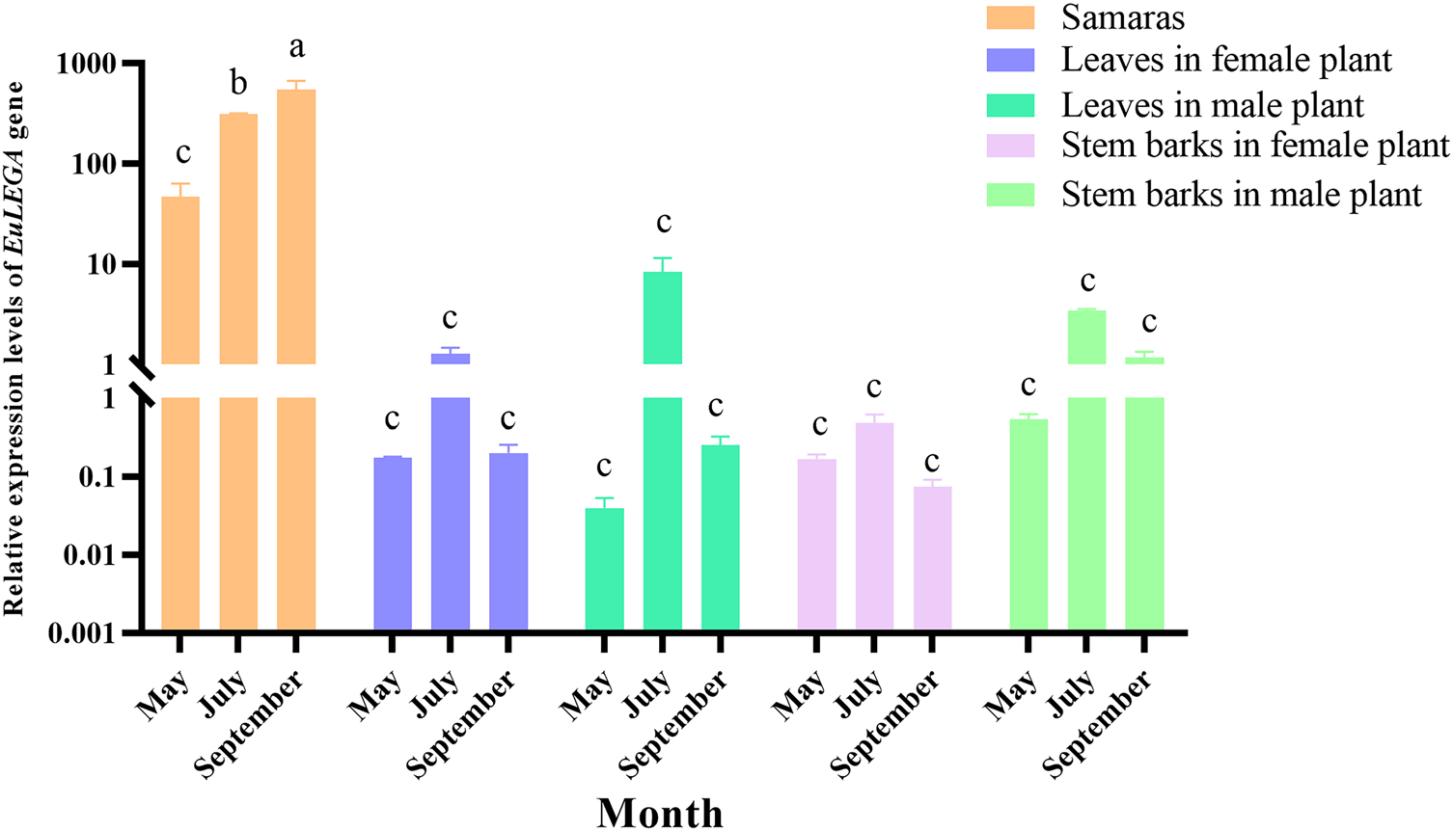
Expression levels of the legumin A gene from *Eucommia ulmoides* in stems, leaves and samaras in May, July and September. Note: The Y-axis gaps adopts a log10 scale pattern, and results obtained from comparing significant differences between all the data separately. Different letters indicate significant differences. *P* < 0.05*.*

Legumin A is a seed storage protein, so the seasonal process of seed formation is valuable for determining whether the *EuLEGA* gene is a synthetic gene for legumin A. The characterization of the expression levels of the *EuLEGA* gene in *E. ulmoides* using real-time fluorescence-based quantitative PCR showed that the *EuLEGA* gene accumulated in the samara at an extremely high rate from April to September and reached a maximum value in September (Fig. 7), accumulating 521.65-fold compared with that in April and then beginning to decline significantly in October. Analysis of the *EuLEGA* gene in samaras, leaves and stem bark in May, July and September revealed that the expression levels of the *EuLEGA* gene in samaras were significantly greater than those in leaves and stem bark in the same month (Fig. 8), indicating that the gene accumulates specifically and extremely quickly in July and September and the expression levels were significantly greater in the samaras than in the leaves and stem bark as a whole. The expression levels of *EuLEGA* gene in leaves of male plants, leaves of female plants, stem bark of male plants, and stem bark of female plants gradually decreased from July to September, indicating that the expression pattern of the *EuLEGA* gene in leaves and stem bark differed from that in samaras, suggesting that the accumulation of the gene in samaras occurred later than in stem bark and leaves.

### Studies on the regulation of *EuLEGA* gene expression

Regulation of the expression of the *EuLEGA* gene was analysed by determining the effects of gibberellin, abscisic acid and methyl jasmonate on the promoter.

#### Cloning of the promoter of the *EuLEGA* gene

Cloning revealed that the promoter sequence was 1892 bp upstream of the *EuLEGA* gene. Four plant expression vectors were obtained, each containing different fragmented promoters.

#### Analysis of the *cis*-acting elements of the *EuLEGA* gene promoter

**Figure 9:**
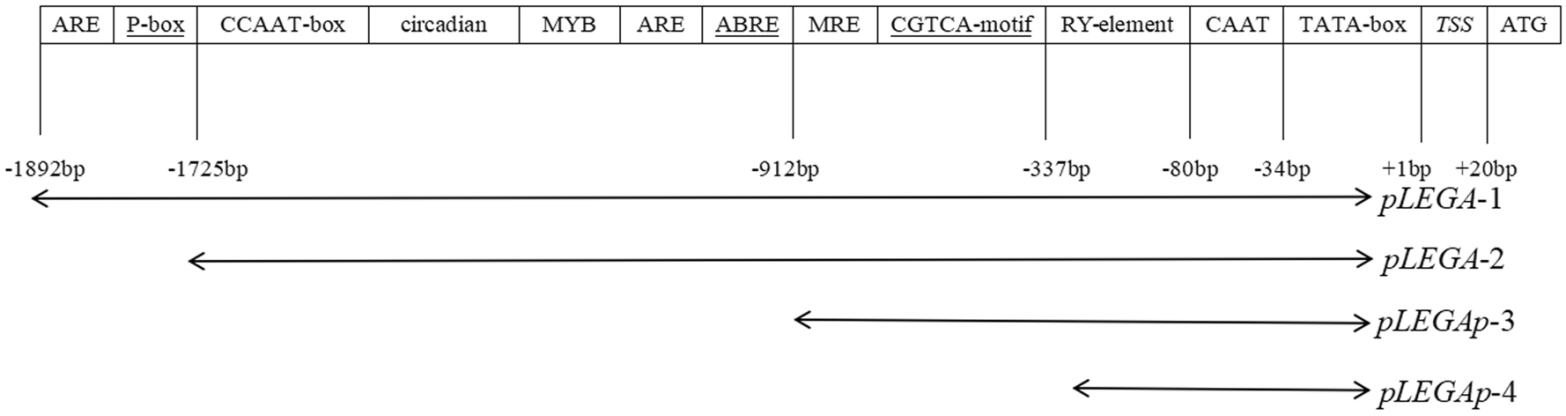
Structure of promoter fragment of the legumin A gene from *Eucommia ulmoides.*

After cloning the promoter sequence located 1912 bp upstream of the *EuLEGA* gene start codon (ATG), we observed that the transcription start site (TSS) was positioned 48 bp upstream of the *EuLEGA* start codon. Moreover, the TATA box and CAAT box were identified at positions 34 bp and 80 bp upstream of the TSS, respectively. Additionally, a total of 10 *cis*-acting elements associated with the light response, one gibberellin response element, one MYBHv1 binding site, one *cis*-acting element involved in the abscisic acid response, two *cis*-acting elements essential for anaerobic induction, four *cis*-acting elements implicated in the MeJA response, and one *cis*-acting element involved in circadian rhythm control and seed-specific regulation were detected. In the context of bioinformatics, we introduced sequences containing partial *cis*-acting elements into the *GUS* gene vector pCAMBIA1381Z. The location of the *cis*-acting element and its 5′ end-truncated structure in pLEGA has been well defined (Fig. 9).

#### Effects of hormone treatment on the expression of the *EuLEGA* gene in *E. ulmoides* seedlings

**Figure 10:**
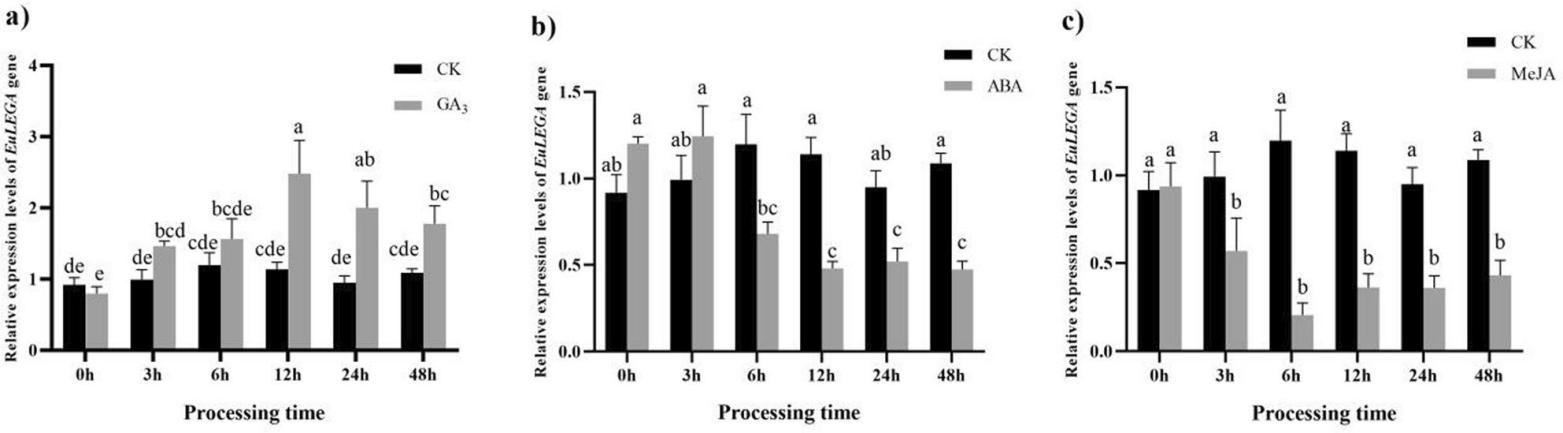
Relative expression of the legumin A gene in *Eucommia ulmoides* seedlings subjected to hormone treatments. Note: (**a**) 100 µM GA_3_ to leaves of *Eucommia ulmoides* seedlings; (**b**) 100 µM ABA to leaves of *Eucommia ulmoides* seedlings; (**c**) 100 µM MeJA to leaves of *Eucommia ulmoides* seedlings. The corresponding solvent (CK) was used in the negative control group. Different letters indicate statistically significant differences. (*P* < 0.05).

After treatment, the leaves of 60-day germinated *E. ulmoides* plants were sprayed with a 100 µM gibberellin (GA_3_) solution, while those in the control group were sprayed with distilled water. The results demonstrated a gradual increase in the expression levels of the *EuLEGA* gene, peaking within 12 h at a magnitude of 3.12-fold before subsequently declining (Fig. 10a). In contrast, the application of 100 µM abscisic acid (ABA) to *EuLEGA*-treated seedling leaves resulted in a significant 1.77-fold reduction in the relative expression of the *EuLEGA* gene within 6 h, followed by a subsequent significant increase of 1.99–2.82-fold over the next 12–48 h compared to that in the control group (Fig. 10b). The expression levels of this gene rapidly decreased after the leaves of *E. ulmoides* plants were sprayed with a 100 µM methyl jasmonate (MeJA) solution, exhibiting a 4.55-fold decrease and reaching its lowest point after 6 h of treatment. Within 24 h, there was a significant increase of 8.45-fold (Fig. 10c). These findings suggested that the *EuLEGA* gene was regulated by GA_3_, ABA, and MeJA.

#### Segmented cloning of promoters

**Figure 11:**
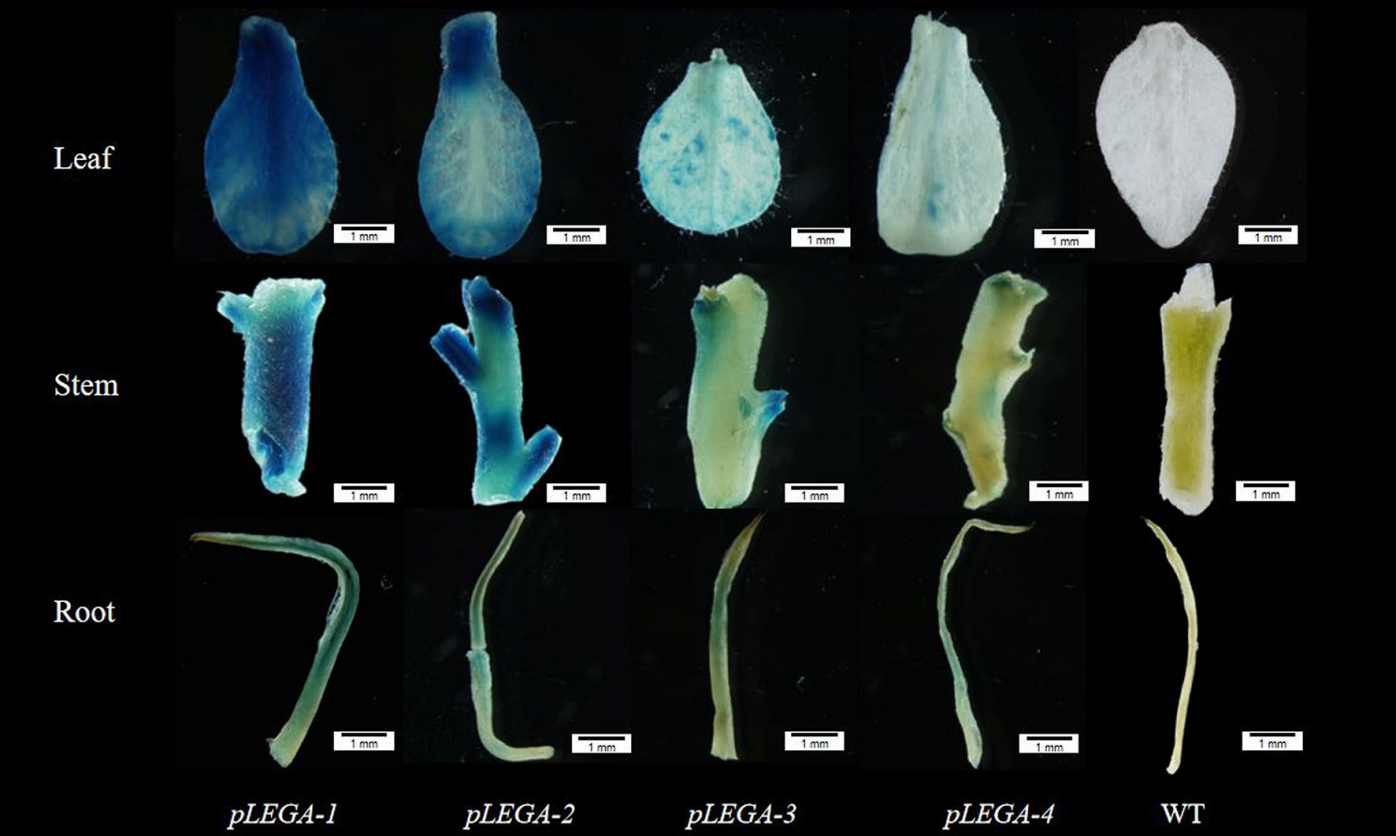
GUS immunostaining of pLEGA-1, pLEGA-2, pLEGA-3, p-LEGA4 transgenic tobacco plants and WT in roots, stems and leaves. Note: Scale bar = 1 mm.

**Figure 12:**
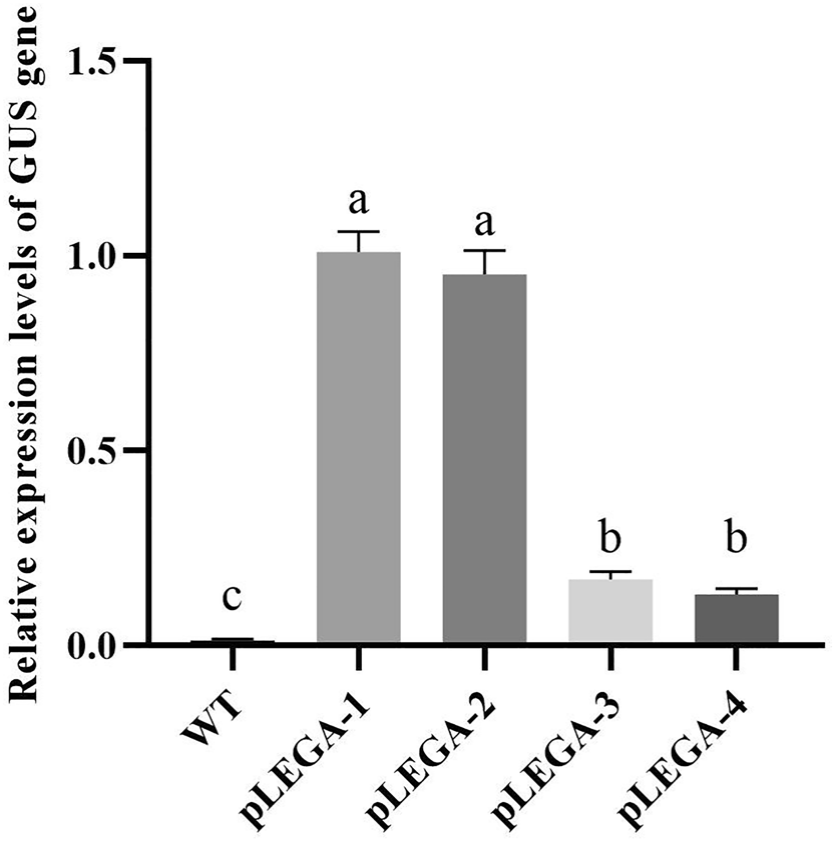
Relative expression levels of *GUS* gene in transgenic tobacco leaves. Note: Different letters indicate significant differences. *P* < 0.05.

The promoter fragment of the missing *cis*-acting element was cloned and inserted into pCAMBIA1381Z-pLEGA; a vector specifically designed for driving *GUS* gene expression (Fig. 9). The expression patterns of pLEGA-1, pLEGA-2, pLEGA-3, and pLEGA-4 in the leaves, stems, and roots of tobacco plants were assessed through GUS histochemical staining. The staining intensity of pLEGA-1 and pLEGA-2 was darker than that of pLEGA-2 with more truncated promoter fragments, whereas pLEGA-3 and pLEGA-4 with increased truncation exhibited lighter staining (Fig. 11). Quantitative analysis of *GUS* gene expression using RT‒qPCR revealed variations among different promoter fragments. Different 5' end truncated promoters displayed distinct abilities to drive *GUS* gene expression. There were no significant differences in *GUS* gene expression between pLEGA-1 and pLEGA-2. Similarly (Fig. 12), the two shorter fragments (pLEGA-3 and pLEGA-4) did not exhibit any significant variation. However, notable distinctions were observed between the longer segments and the shorter segments.

#### Analysis of *GUS* expression levels after hormone treatment

**Figure 13:**
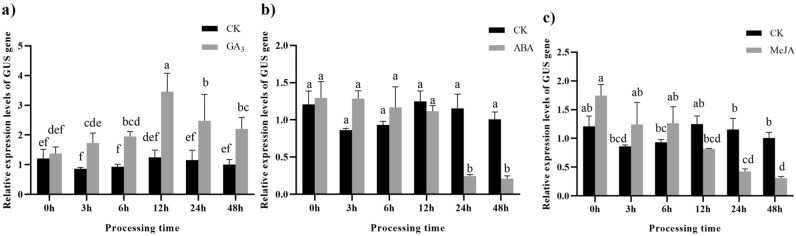
Relative expression levels of *GUS* gene driven by pLEGA-1 under different treatments. Note: (**a**) Transgenic tobacco leaves were sprayed with 100 µM GA_3_; (**b**) Transgenic tobacco leaves were sprayed with 100 µM ABA; (**c**) Transgenic tobacco leaves were sprayed with 100 µM MeJA. The corresponding solvent (CK) was used in the negative control group. Different letters indicate significant differences. *P* < 0.05.

Transgenic tobacco plants harbouring the CCTTTTG promoter region of pLEGA-1 were subjected to treatment with 100 µM GA_3_. Using 0 h as the baseline, the relative expression of the *GUS* gene exhibited a significant upwards trend as the treatment duration increased, reaching a 2.5-fold increase at 12 h and subsequently decreasing at 24 h (Fig. 13a). Following treatment with 100 µM ABA for 24–48 h, there was a substantial reduction in the relative expression of the *GUS* gene in pLEGA-1 transgenic tobacco leaves by approximately 5.27–6.16-fold (Fig. 13b). Moreover, upon treating pLEGA-1 transgenic tobacco leaves with 100 µM MeJA there was a gradual decrease in the relative expression level of the *GUS* gene with increasing treatment time, indicating that MeJA significantly inhibited its expression. Compared to the reference point (0 h), after MeJA treatment for durations of up to 24 h and 48 h, *GUS* was downregulated by approximately 4.11- and 5.66-fold, respectively (Fig. 13c).

**Figure 14:**
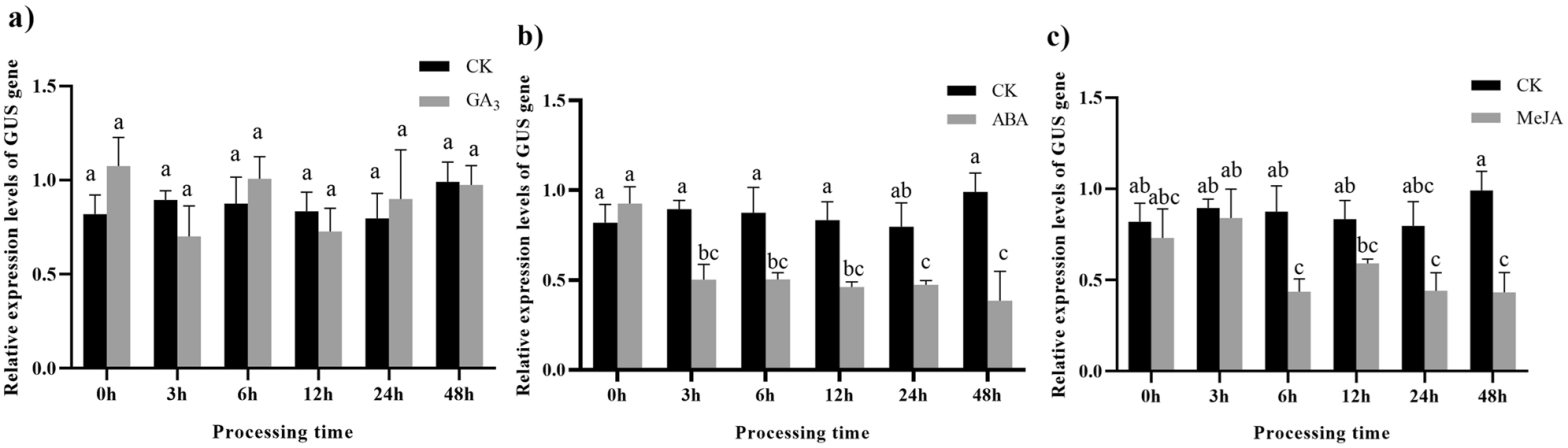
Relative expression levels of *GUS* gene driven by pLEGA-2 under different treatments. Note: (**a**) Transgenic tobacco leaves were sprayed with 100 µM GA_3_; (**b**) Transgenic tobacco leaves were sprayed with 100 µM ABA; (**c**) Transgenic tobacco leaves were sprayed with 100 µM MeJA. The corresponding solvent (CK) was used in the negative control group. Different letters indicate significant differences. *P* < 0.05*.*

After treatment with 100 µM GA_3_, there was no significant difference in the relative expression of the *GUS* gene in pLEGA-2 transgenic tobacco plants lacking CCTTTTG promoter elements (Fig. 14a). Within 3 h following treatment with 100 µM ABA, a significant decrease of 1.84-fold was observed in the relative expression of the *GUS* gene, which then remained stable (Fig. 14b). Conversely, upon treating pLEGA-2 transgenic tobacco leaves with 100 µM MeJA for 6 h, a sustained decrease of 1.67-fold in *GUS* gene expression was observed (Fig. 14c).

**Figure 15:**
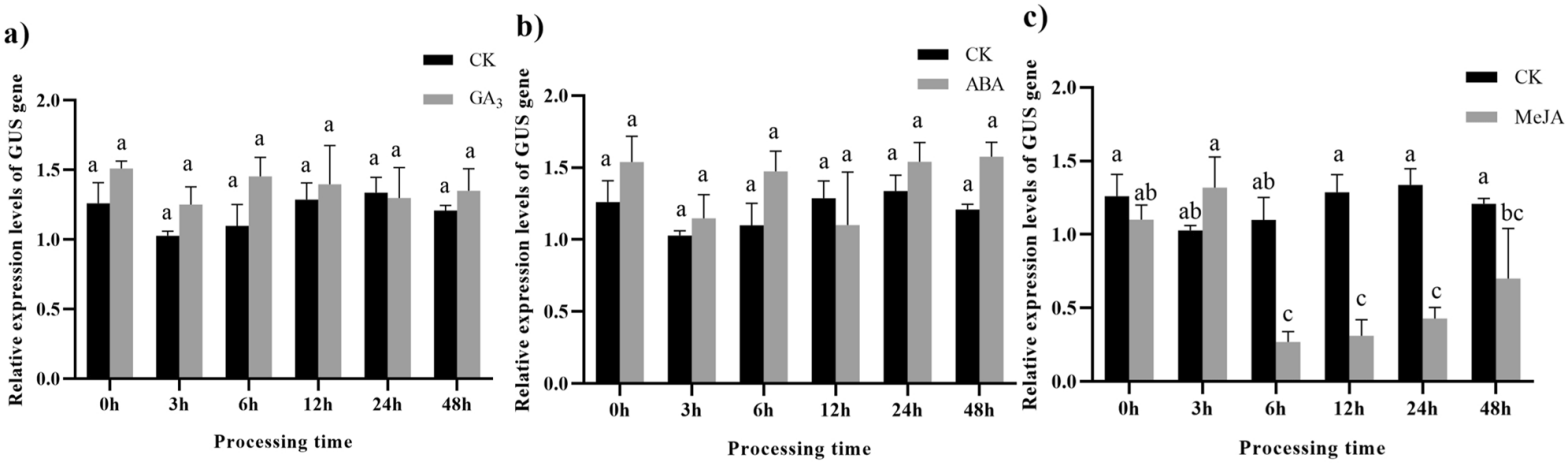
Relative expression levels of *GUS* gene driven by pLEGA-3 under different treatments. Note: (**a**) Transgenic tobacco leaves were sprayed with 100 µM GA_3_; (**b**) Transgenic tobacco leaves were sprayed with 100 µM ABA; (**c**) Transgenic tobacco leaves were sprayed with 100 µM MeJA. The corresponding solvent (CK) was used in the negative control group. Different letters indicate significant differences. *P* < 0.05.

After treatment with 100 µM GA_3_ and ABA, no significant difference in the relative expression of the *GUS* gene was detected in pLEGA-3 transgenic tobacco leaves lacking the CCTTTTG and ACGTG promoter elements (Fig. 15a,b). Following treatment of pLEGA-3 transgenic tobacco leaves with 100 µM MeJA, *GUS* gene expression decreased to a minimum of 4.10-fold within 6 h and gradually recovered to control levels (Fig. 15c).

**Figure 16:**
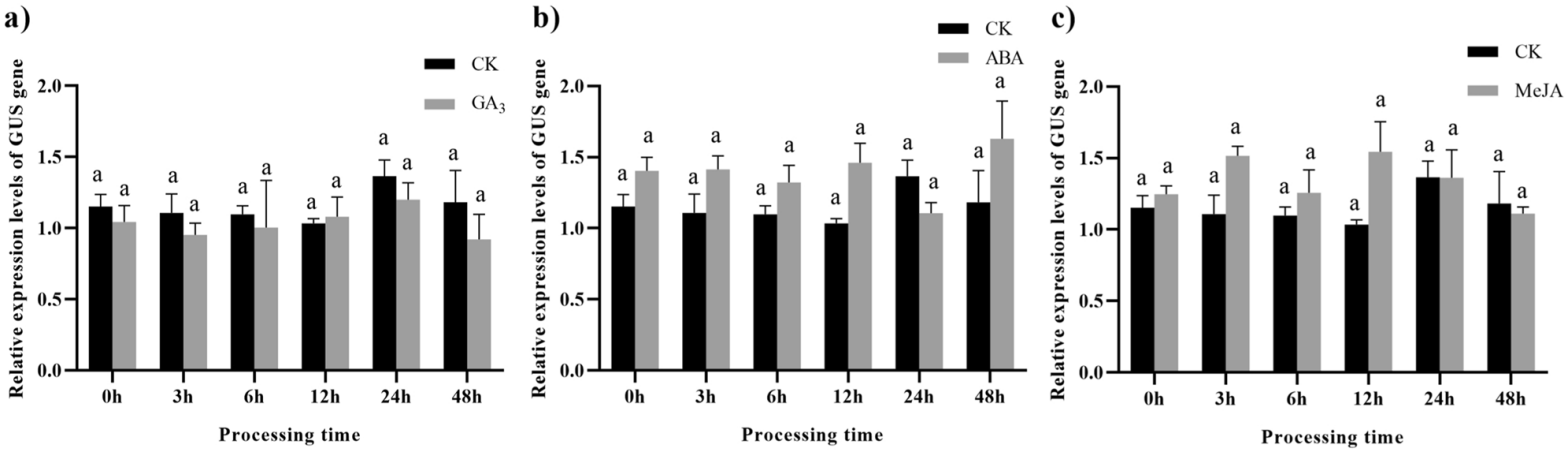
Relative expression levels of *GUS* gene driven by pLEGA-4 under different treatments. Note: (**a**) Transgenic tobacco leaves were sprayed with 100 µM GA_3_; (**b**) Transgenic tobacco leaves were sprayed with 100 µM ABA; (**c**) Transgenic tobacco leaves were sprayed with 100 µM MeJA. The corresponding solvent (CK) was used in the negative control group. Different letters indicate significant differences. *P* < 0.05.

After treatment with 100 µM GA_3_, ABA, and MeJA, the leaves of pLEGA-4 transgenic tobacco plants without CCTTTTG, ACGTG, and CGTCA promoter elements showed no significant difference in the relative expression of the *GUS* gene (Fig. 16a–c).

## Discussion

The CDS region length of the *EuLEGA* gene was 1287 bp, encoding a total of 428 amino acid residues, with an approximate molecular weight of 48.04 kDa. Through analysis, it was determined that this gene belongs to a superfamily characterized by the presence of the Cupin_RmIC-like conserved domain. This superfamily harbours both the RmIC enzyme, which is responsible for catalysing rhamnose synthesis, and the conserved legumin domain^29^. Phylogenetic analysis revealed a close relationship between EuLEGA and the legumin proteins found in tobacco, sesame, and other seed plants (Fig. 1). Previous studies have demonstrated that the sequence homology of a single legumin 11S ranges from 50 to 95%^39^, which aligns with our homology analysis of the EuLEGA protein. Consequently, we provisionally designated this gene the legumin A gene (*EuLEGA*).

The storage proteins in legumes predominantly accumulate in the cotyledons of seeds, serving as vital nutrients for seed germination and seedling growth. These proteins exhibit nonenzymatic activity and play a pivotal role in the maintenance of seed development^40,41^. The content of legumin significantly increased in both the transgenic tobacco and transgenic *E. ulmoides* plants (Figs. 2 and 4). While the content of rhamnose did not significantly change in the tobacco plants, it significantly increased in the *E. ulmoides* plants (Figs. 2 and 4). Following genetic transformation with the interference vector, there was no significant change in the content of rhamnose in transgenic *E. ulmoides*; however, there was a notable decrease in the content of legumin (Fig. 4). Our determination of the content of legumin in transgenic tobacco seeds revealed a significant increase in the content of legumin in transgenic tobacco seeds, which further demonstrated that the *EuLEGA* gene is associated with legumin synthesis (Fig. 5). Therefore, it can be inferred that the *EuLEGA* gene is intricately associated with the increase in leguminA content, suggesting its potential role in encoding leguminA. Regarding the formation of internal cell structures, it is plausible that the EuLEGA protein plays a role in the development of protein storage vacuoles (PSVs)^42^. Rhamnose is synthesized via distinct pathways in plants and bacteria. In bacterial cells, the biosynthesis of L-rhamnose involves a cascade of four enzymatic reactions: glucose-1-phosphate thymidyltransferase (RlmA), dTDP-d-glucose 4,6-dehydratase (RmlB), RmlC, and dTDP-6-deoxy-l-lyxo-4-hexulose reductase (RmlD). Notably, RmlC acts as the pivotal catalyst responsible for converting deoxythymidine diphosphate (dTDP) to the l-form of rharose^43,44^. In plants, UDP-rhamnose is synthesized by a multifunctional rhamnose synthetase (RHM), and the final two steps can be catalysed by a bifunctional UER enzyme^44^. After the genetic transformation of *E. ulmoides*, there was a significant increase in the content of rhamnose, suggesting that the presence of the *EuLEGA* gene could further increase rhamnose levels in *E. ulmoides*. It is plausible that seed mucous formation and the positive regulation of other transcription factors may involve rhamnose synthase (RHM), which synthesizes UDP-rhamnose^45^. However, the exact causes and underlying mechanisms remain unclear.

Mature 11S globulins are composed of hexameric complexes, wherein each morph subunit is composed of a polypeptide connected through acidic and basic disulphide bonds^46,47^. The synthesis of each pair of peptides begins with a single precursor, which subsequently assembles into a 9S trimer in the endoplasmic reticulum upon attaining a higher-order structure. Eventually, these precursors reach maturity within the vacuole^48,49^. In their study on the synthesis of grain seed storage proteins, Kawakatsu et al.^42^ reported that globulins undergo processing through the endoplasmic reticulum and Golgi before entering the vacuole, where they form a matrix-like structure surrounding it. In plant cells, the continuous catalysis of L-rhamnose is facilitated by three glycosyltransferases, namely RHM and UER, utilizing UDP-glucose as a substrate. Primarily localized in the cell wall matrix, this process has been reported by Jiang et al.^44^. The bioinformatics analysis in this study predicted the subcellular localization of the protein to be within the vacuole. To validate this prediction, we conducted subcellular localization experiments using *A thaliana* protoplasts and confirmed the presence of the protein in both the cytoplasmic and vacuolar membranes (Fig. 6). This finding is consistent with the previously reported location of most legumin plants in vacuoles. These findings suggest that the protein may undergo intracellular transport and subsequently exert its function on the vacuole membrane. Rhamnose is an important part of the cell wall and is involved in the synthesis of pectin polymers and cell wall glycoproteins in plant cell walls^50^ (Supplementary information). The results of this study are inconsistent with the results of rhamnose localization in the cell wall. Thus, it was further demonstrated that this gene encodes legumin.

Previous studies have demonstrated that rice seeds undergo the synthesis and deposition of SSPs within endosperm storage organelles during maturation, serving as a crucial nitrogen source for subsequent seedling germination^51^. The expression of the storage protein gluten in wheat seeds has been demonstrated to be limited to the endosperm of transgenic tobacco plants, with no detectable expression observed in other tissues^52^. However, our analysis of *GUS* expression patterns revealed that the promoter of the *EuLEGA* gene exhibits broad tissue specificity in driving *GUS* gene expression in diverse tissues. This finding suggests that the promoter of the *EuLEGA* gene can drive gene expression in any tissue (Fig. 11). However, whether this gene is specifically expressed in seeds warrants further study. By assessing the *GUS* gene expression levels of progressively truncated promoter fragments, we found that the core element of the promoter of the *EuLEGA* gene is located between − 912 and − 1725 bp. The upstream regions did not exhibit any significant impact on *GUS* expression patterns (Fig. 12). Furthermore, several studies have suggested that upstream regions might influence the transcription rate^53^. However, additional experimental investigations are required to ascertain the extent of this impact on the transcription rate. The expression levels of *GUS* in transgenic tobacco leaves were significantly upregulated upon treatment with GA_3_, while they were significantly downregulated following treatment with ABA and MeJA (Figs. 13, 14, 15 and 16). These findings are consistent with those observed in *E. ulmoides* seedlings treated with the same hormones (Fig. 10), suggesting that GA_3_ promotes *EuLEGA* gene expression whereas ABA and MeJA inhibit *EuLEGA* gene expression. Conversely, stress can regulate gene expression and many *cis*-acting elements at promoters play important roles in transcriptional regulation: plant resistance to stress can be improved by regulating the expression of downstream genes^54,55^. Adverse stress is an important factor affecting the germination and growth of *E. ulmoides* seeds. Our experiments revealed that this promoter is an important element in the regulation of transcription processes and largely determines the expression levels of downstream genes, which is consistent with the findings of previous studies^56,57^.

Legumin, a globulin belonging to the same class as pea globulin, was initially discovered in legumes as a seed storage protein in angiosperms and gymnosperms^58^. Therefore, the process of seasonal seed formation is important for exploring whether the *EuLEGA* gene is a synthetic gene of legumin A. Analysis of the expression characteristics of the *EuLEGA* gene revealed significant temporal changes in its expression levels during the period from April to October in adult *E. ulmoides* samaras. Specifically, its expression levels rapidly increase between May and September, peaking in September (Fig. 7). This phenomenon can be attributed to the low GA_3_ accumulation from April to May and the subsequent high GA_3_ accumulation from May to September, resulting in the upregulation of the *EuLEGA* gene. Additionally, the decreased expression levels of this gene after September may be associated with the accumulation of ABA and MeJA. This study is consistent with the results of Lei et al.^59^ on the gradual accumulation of 11S in samaras. Importantly, the *EuLEGA* gene exhibited significantly greater expression in samara tissues than in stem and leaf tissues (Fig. 8), further validating its role as a legumin A-encoding gene. Studies have shown that abscisic acid can reduce the production of reactive oxygen species (ROS) in rice seeds to inhibit seed germination, and a potent reactive oxygen species scavenger, APX, controls GA_3_ synthesis to inhibit seed germination by reducing ROS and ASC^60^. This study revealed the mechanism of ABA and GA_3_ during seed germination. In conclusion, through in-depth studies of the expression characteristics of the *EuLEGA* gene and its seasonal expression patterns in fruits, we were able to better understand the role of legumin in plant growth and development and how it influences plant responses to environmental changes. These findings have important implications for the development of new crop breeding strategies to improve crop yield and quality.

## Materials and methods

### Ethics statement

All the methods were performed in accordance with relevant guidelines and regulations.

### Biomaterials and reagents

*E. ulmoides* seeds (Huazhong No. 12) were purchased from our laboratory from Hengsheng Seedling Seed Company (Shanxi, China). Tissues of *E. ulmoides* (including roots, stems, leaves and fruits) were collected from the Plant Germplasm Resource Planting Base of Guizhou University in 2022 (Guizhou, China). Tobacco (*Nicotiana Xanthin*.) seeds, the plant expression vectors pCAMBIA1301-35S-NOS and pCAMBIA1300-35S-*EGFP*-NOS, the *Escherichia coli* (*E. coli*) strain DH5α, and *Agrobacterium tumefaciens* GV3101 were obtained from our laboratory (Guizhou, China). The expression vector pCAMBIA1381Z was purchased from Wuhan Transduction Biology Laboratory (Wuhan, China). A plant RNA extraction kit (Commodity No.: CW0559S) was purchased from Kangwei Century Biotechnology Company Limited (Co., Ltd.) (Beijing, China). A reverse transcription kit (commodity no.: M16315 + M16325), a high-fidelity enzyme (Commodity No.: F630S), and a Taq enzyme (Commodity No.: 10966083) were purchased from Thermo Fisher (Waltham, Massachusetts, USA). Real-time fluorescence-based quantitative PCR (Commodity No.: A311-10) enzymes were purchased from Genstar (Beijing, China), and relevant biological reagents for tissue culture, such as MS media, sucrose, agar, GA_3_, ABA, MeJA, NAA, 6-BA, Timentin, and hygromycin (Commodity No.: M8521, S8271, A8190, G8040, A8060, M8640, N8010, A8170, T8660, H8081), were all purchased from Solarbio (Beijing, China). The rhamnose standard (commodity no.: B21931-100 mg) was purchased from Shanghai Yuanye Biotechnology Co., Ltd (Shanghai, China). The bean globulin detection kit (Commodity No.: GLB11 s) was purchased from Shanghai Yuanxin Biotechnology Co., Ltd (Shanghai, China).

### Gene cloning and vector construction

Total RNA was extracted from the young leaves of *E. ulmoides*, reverse transcribed into cDNA and cloned and inserted into the *EuLEGA* gene. The PCR product of T-vector cloning was sent to Bioengineering (Shanghai, China) Co., Ltd. for sequencing, and the correct sequences were inserted into the linearized empty vector pCAMBIA1301-35S-NOS by homologous recombination to obtain the expression vector pCAMBIA1301-EuLEGA-NOS. The *EuLEGA* gene in pCAMBIA1301-35S-*EuLEGA*-NOS was cloned and the stop codon was removed by attachment to the linearized plant expression vector pCAMBIA1300-*EGFP-*NOS by homologous recombination to obtain the fusion expression vector pCAMBIA1300-35S-*EuLEGA*-*EGFP*-NOS.

Thermo Fisher’s online software Invitrogen Block-iT RNAi Designer (https://origin-k8s-prodb.cloudprod.thermofisher.com/rnaiexpress/) (Waltham, Massachusetts, USA) was used to design interference fragments (the 425–725 bp sequence of the *EuLEGA* gene). A search through the *E. ulmoides* whole-genome database revealed introns. The intron fragment of *E. ulmoides* Nop 53 was selected to connect the sense and antisense strands to form a stem‒loop structure, and the RNAi expression vector pCAMBIA1301-35S-*EuLEGA* RNAi-NOS was obtained.

### Bioinformatics analysis

The conserved structural domains of the EuLEGA were analysed using the online software Search for Conserved Domains (https://www.ncbi.nlm.nih.gov/Structure/cdd/wrpsb.cgi) in NCBI (Bethesda, Maryland, USA). The physicochemical properties of the EuLEGA were analysed using the online software ExPASy ProtParam (https://web.ExPASy.org/protparam/) (Geneva, Switzerland). The transmembrane structural domains and subcellular localization of the protein were predicted using the TMHMM (http://web.ExPASy.Org/protparam/) (Geneva, Switzerland) and WOLFPSORT (http://web.ExPASy.org/protparam/) (Geneva, Switzerland) programs. Enzymatic site analysis was performed using SnapGene Version 6.0.2 IP: 192.168.4.30. (https://snapgene.software.informer.com/) (Boston, USA) software and primer design was carried out using the Primer Designing Tool of NCBI (https://www.ncbi.nlm.nih.gov/tools/primer-blast/) (Bethesda, Maryland, USA). The genetic information of *Nicotiana sylvestris* (Accession No.: XP_009763034), *Cynara cardunculus* var. Scolymus (accession: XP_024981846), *Nicotiana attenuata* (accession: XP_019242883), *Lactuca sativa* (accession: XP_023733339), *Solanum tuberosum* (accession: XP_006351673), *Dorcoceras hygrometricum* (accession: KZV32997), *Erythranthe guttata* (accession: XP_012828922), *Sesamum indicum* (accession: XP_020549903), and *H. brasiliensis* (accession: XP_021676679) was obtained from the NCBI website (https://www.ncbi.nlm.nih.gov/) (Bethesda, Maryland, USA). Amino acid in different species were compared using DNAMAN version 9 (Lynnon Biosoft, USA).

### Genetic transformation of plant expression vectors

The plant expression vectors pCAMBIA1301-35S-*EuLEGA*-NOS and pCAMBIA1301-35S-*EuLEGA* RNAi-NOS were genetically transformed into tobacco and *E. ulmoides* by using *A. tumefaciens* (GV3101). The leaf disc method was used for the genetic transformation of tobacco, and the hypocotyl was infected via genetic transformation of *E. ulmoides*. After infection, dark culture, screening and subculturing, resistant tobacco and *E. ulmoides* buds were obtained. The positive plants were identified with GUS staining. The following experiments were carried out after the roots of the positive plants were removed.

### Determination of rhamnose content

The samples were processed according to previous methods^61^ with modifications. Approximately 1 g of transgenic, RNAi, and WT *E. ulmoides* bud was accurately weighed in a 15 mL clear centrifuge tube, 5 mL of distilled water was added, and ground into a homogenate. The mixture was fully oscillated on an oscillator, mixed for 1 min, ultrasonicated at 50 °C for 1 h, removed after cooling, and centrifuged at 4000 r/min for 10 min. The supernatant was passed through a 0.45 μm filter membrane for analysis with liquid chromatography. The test conditions were as follows: RID detector, NH_2_ chromatographic column (250 × 4.6 mm; 5 μm), column temperature, 40 °C; flow rate, 1 mL/min; injection volume, 20 μL; and mobile phase, acetonitrile:water = 75:25 (v/v). Isocratic elution was performed.

### Determination of legumin A content

Sample processing was carried out according to the method of Suchkov et al.^62^ for the isolation and purification of 7S and 11S globulins with modifications. The processed samples were tested with an enzyme-linked immunosorbent assay (ELISA) using a double antibody one-step sandwich method according to the manufacturer’s instructions. The specimen, standard and HRP-labelled detection antibody were added sequentially to the coated microtiter wells precoated with 11S globulin antibody, after warming and thorough washing. The color is developed with the substrate TMB, which is converted to a blue color by peroxidase catalysis and to finally a yellow color in the presence of acid. The shade of color is positively correlated with the 11S globulin in the sample. The absorbance (OD) was measured at 450 nm using an enzyme marker, and the sample concentration was calculated.

### Subcellular localization

Cell-PLoc 2.0 (Shanghai, China) was used to predict the subcellular localization of the EuLEGA protein online. *A. thaliana* protoplasts were prepared with reference to^63^, *A. thaliana* protoplasts were transformed with the pCAMBIA1300-35S-EuLEGA-GFP-NOS plasmid at 22.5 °C in a water bath, and GFP fluorescence was observed with a laser confocal microscope to determine the position of the EuLEGA protein in the cells.

### Real-time qPCR

Total RNA was extracted and then reverse transcribed to cDNA using an RNA extraction kit and real-time qPCR was used to detect the relative expression of the *EuLEGA* gene. The data were analysed using the Livak method.

### Promoter cloning and *cis*-acting element analysis

Based on the *E. ulmoides* whole-genome annotation database constructed in the early stage of this study, primers were designed for the upstream DNA sequence of the *EuLEGA* gene. The upstream 1912 bp sequence was amplified by PCR, and the upstream 1892 bp sequence of the *EuLEGA* gene was analysed using PlantCARE (http://bioinformatics.psb.ugent.be/webtools/plantcare/html/) (Ironmould Lane, Brislington, Britain) online analysis software to preliminarily determine the location of the *cis*-acting element. Moreover, plant expression vectors driving *GUS* gene expression were constructed using upstream sequence fragments of different lengths, including (1) pCAMBIA1381Z pLEGA-1::*GUS* (− 1 to − 1892 bp), which includes a CGTCA motif, P-box, ABRE, and circadian *cis*-acting elements involved in circadian rhythm control; (2) PCAMBIA1381Z pLEGA-2::*GUS* (− 1 to − 1395 bp), which includes drought-responsive MYB elements, ARE responsive elements necessary for anaerobic induction, and photoresponse elements; and (3) PCAMBIA1381Z pLEGA-3::*GUS* (− 1 to − 690 bp) and (4) pCAMBIA1381Z pLEGA-4::*GUS* (− 1 to − 332 bp), both of which include transcription start sites, CAAT boxes, TATA boxes, and RY-element *cis*-acting elements involved in seed-specific regulation.

*E. ulmoides* seedlings grown for 60 days after seed germination were used as materials. The plants were treated with 100 µM GA_3_, ABA or MeJA. Samples were taken at six time points (0 h, 3 h, 6 h, 12 h, 24 h, and 48 h) after treatment to detect the expression levels of the *EuLEGA* gene.

Similarly, transgenic tobacco plants genetically transformed with *GUS* gene expression vectors constructed with *EuLEGA* gene upstream promoters of different lengths were treated with 100 µM GA_3_, ABA, or MeJA respectively. At six time points after treatment (0 h, 3 h, 6 h, 12 h, 24 h, and 48 h) the expression levels of the *GUS* gene were detected.

### Statistical analysis

The bar charts were made using GraphPad Prism 8.0.1 (GraphPad Company, Boston, MA, USA). ANOVA with LSD and Duncan test were used to analyze the significant difference in gene expressions in SPSS version 22.0 (IBM, Armonk, NY, USA). The *P* value of 0.05 was considered to be significant.

### Supplementary Information

Supplementary Information.

## Data Availability

The *E. ulmoides* transcriptome database has been successfully uploaded to the NCBI database (https://www.ncbi.nlm.nih.gov/datasets/genome/GCA_016647705.1/). The data that support the findings of this study are available from the corresponding author, [De-Gang Zhao], upon reasonable request.
