# Supplementary material for: Cloning and functional characterization of the legumin A gene (EuLEGA) from Eucommia ulmoides Oliver
Source: Sci Rep. 2024 Jun 19;14:14111. doi: 10.1038/s41598-024-65020-5 (PMC11187137; doi:10.1038/s41598-024-65020-5)
Supplement: Supplementary file 1 — Supplementary Information. [file 41598_2024_65020_MOESM1_ESM.pdf]

Additional information

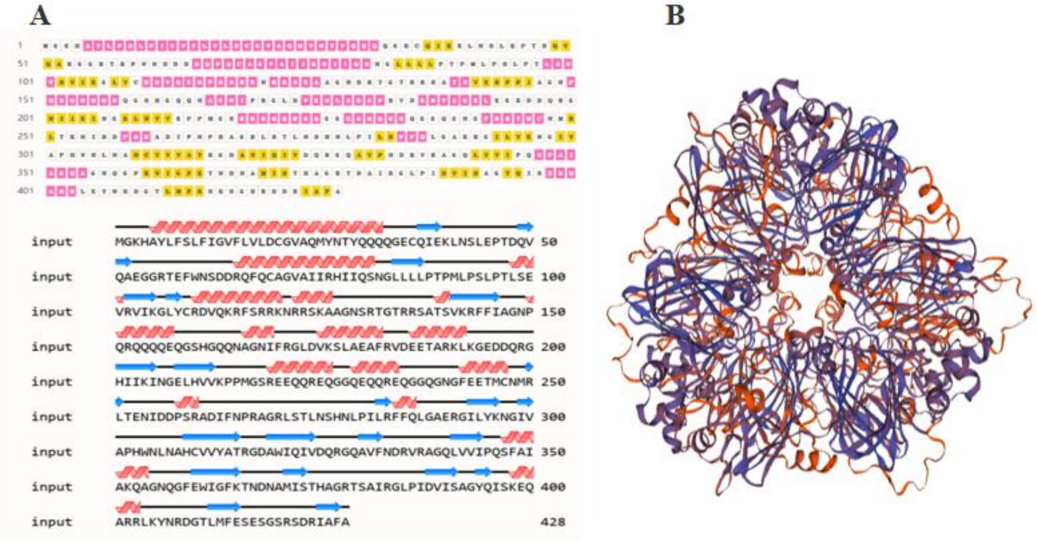

Supplementary Figure 1 Schematic diagram of the protein structure encoded by *EuLEGA*. A) The secondary structure of *EuLEGA* encoded proteins; B) The tertiary structure of *EuLEGA* encoded proteins.

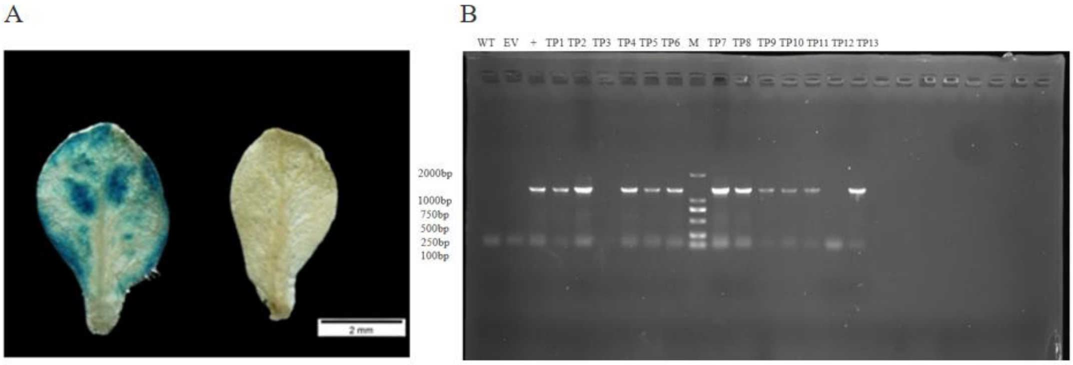

Supplementary Figure 2 Identification of pCAMBIA1301-35S-EuLEGA-NOS genetically transformed tobacco. A) GUS identification of transgenic tobacco leaves; B) Direct PCR identification of transgenic tobacco leaves. Scale bar = 2mm.

CAGTTTCCATGTGAGAGAGCCTGGAAATATTTTATTGACCATCTCCAATTGGACGTTCAAGAAGAAGA

AGGTCTTCTATAGATTTAAAACCAAGCCCTTTGTGCCCTTATACACCACCTCCTTCAAGTGATGATTGAT

ARE

GTCTTTTTTTCTTTTGGAAAAAAAAAAAAACCTTTTGATCAAAGTGAAATAATATTTTAAAATTCACAAG

P-box

AAAAGTGAATTTCGTAGTATTTTATTTGATTAGTGAAATATTTTTCTCTTCAAAAGTCTAGGGTTTTGTTT

TGGGCAACGGGGTTTATAGGGTTTTGATCCAGGTAGCGGCGGTGTCCAGAAGGAACTCAAGTCCCTCT

CCAAT-box

GAATTTTATGAAAATGTTTACTGTTAGAATAATTTAGGGACGAAATATTGAGAGAACATGAAATGATTAT

ATTGATATATGAAAAATGTACAATAAACAAAGATATATAAACAAAGAGTCTATATATAAAGTATAACTAA

circadian

AGTTTTTTTTAGTAGGAGTAAGGTTTGAACTCCTCCCCAATCAATTTTTTGGATCCGCAAAATTCGATC

GAATCAATTAATTCTAACTCAACCAACAATATGCTACATATTTTTTTTAAGAGACATAATAATAATATTGA

MYB

ATGTACGATATAAAAGGTAAATTTGGAAATTGAATAAGTACTCTTCCAATAATAGAATGATGACAAATAG

GTTTTTTTTTTTTTTTTTTTTTTTTTTTTTTTTTTTTTTTAAACGAAGGGGGGGTTCGGGAAAGTTTACCCCC

CCCCCACCTTTCCCCTTCCCCATGCGATTTTATGGGCCCCCCCCCCCTACTTCGACTTGATGCTTTCAAAT

TTAAATTCAAAATTTAATTCATGAAGCGCTCCCCATGAAAGCCGAATACAGATTGACCAAGAAAATAGA

GTATCTAACCTTACAAACCAGACTGACAAAAGTCTAAAATTTAGGGTTTAAGATACTGATTCAAGAATG

ARE

GAGGTACGTGTGTAGAAAGGGACTCGACTCTTCCTGAACTTTGATCGTCTCATAAGAAATTGGTGGAC

ABRE、I-box、AE-box、G-box、Box4、MRE

TTTGATCGTCTCAACGTCTTTAAGATCGTTTGGGTGAACTATTTGAAGAAAGAACTTAATTTTTTTTAAA

TCAAATTCTTTCTTCAACTAAAATACGCACTCTTAACCTAATTTGAACCTTCCCAAGTGGATCCATCATC

MRE

AAATCAAAATAATCGACGGTTGAGGAAGTGGGGGATCATAGCATGTGATCCCATTGGCAACAACCTTC

TAAAAAATACATGCAAAAAACATCCAAAAAAAAAATAAAAATAATAATAATAAAAAAGGGATCGGTGAA

CCGTTCACAATTTAAAATTTTTATCTTGAAATATACACACTACACAGTAATTTTTTTTAAGAATTTCAAA

GTTAATCTCTTGTATGAAAATTGTTTGAATAATTTGGAGGCGAAATATTTAGAGAACATGAGAGAGATG

TACTGATTTATAAAAAAAAAATACAATTACATAGATATACAAGGAGTCTATATATAAGAGGTAATTAGAGT

AGTCTTGATAAGGTTAGAGTTTGATTTCGTCAGTGTGACTAAGGTTTAATATAGTGGGCAATGTTCATAT

CGTCA-motif

ACATATATTAATAAAAATTAAAATAGTTGTAGTAATTGAATTGAGGGAATAATAAATTACAAAATTTAAA

ATAATCCTAAAAAAATCCCAAAAAAAAAAACAAATTATGAGAAACAAACAAAAGAATTGAGTTGTTG

CATATGGGAGAAGAAGGGCCACCTGCCCTAAATGCATGTCTAGGTGTAAGAAAGTAGCCATGCATGAC

RY-element CGTCA-motif

GCAACATGGCAATGCCCTTCTCATTATGTGTCACTCCCATACCCACCCTTTACACCCTATAAATACCCTC

CAAT-box TATA-box

TGCGTTACCTACTCGCCACCTCACACTCAGAATCACTTCCCCTCTCTCTTCTTTTCTCTCTCTAGAC

TSS

Supplementary Figure 3 Promoter sequence of *EuLEGA* gene and its cis-regulatory element position

Supplementary Table 1

List of primers related to *EuLEGA* gene

Primer name	Primer sequence (5’—3’)
CDS cloning-F	agctcggtacccgggATGGGTAAGCACGCTTACTTGTTC
CDS cloning-R	agatcttcgtcgactTCAAGCAAACGCAATCCTGTC
Subcellular localization-F	cgaacgatagccatggtaccATGGGTAAGCACGCTTACTTGTTC
Subcellular localization-R	cctgcggccgcgccggatccAGCAAACGCAATCCTGTCG
RNAi-Intron-F	TGTATCTAGCATACTACGCATG
RNAi-Intron-R	GCGTAAGCCACAATTAAAGCG
pLEGA-1-F	atgaccatgattacgaattcCAGTTTCCATGTGAGAGAGCCTG
pLEGA-2-F	atgaccatgattacgaattcGTAGGAGTAAGGTTTGAACTCCTC
pLEGA-3-F	atgaccatgattacgaattcTTGAGGAAGTGGGGGATCATAG
pLEGA-4-F	atgaccatgattacgaattcGTGTGACTAAGGTTTAATATAGTGGGC
pLEGA-R	tggctgcaggtcgacggatccGAAGTGATTCTGAGTGTGATGTGG
Eu-qRT-PCR-F	GCCTTCTGCTCCTTCCTACA
Eu-qRT-PCR-R	CTTGCTCCTCCGATTCTTGC
EuActin-F (qRT-PCR)	GTGTTATGGTTGGGATGGG
EuActin-R (qRT-PCR)	TGCTGACTATGCCGTGTTC
GUS-F (qRT-PCR)	ACTGCTGCTGTCGGCTTTC
GUS-R (qRT-PCR)	GCACCTTGCGGACGGGTAT
β-actin-F (qRT-PCR)	GATCTTGCTGGTCGTGATCT
β-actin-R (qRT-PCR)	ACTTCCGGACATCTGAACCT

Supplementary Table 2

Some cis-regulatory elements of *EuLEGA* gene promoter

Cis-regulatory name	Sequence	Quantities	Function
I-box、AE-box、G-box、Box4、MRE	TACGTG	10	cis-regulatory regulatory element involved in light responsiveness
P-box	CCTTTTG	1	gibberellin-responsive element
CCAAT-box	CAACGG	1	MYBHv1 binding site
ABRE	ACGTG	1	cis-regulatory element involved in the abscisic acid responsiveness
ARE	AAACCA	2	cis-regulatory regulatory element essential for the anaerobic induction
CGTCA-motif	CGTCA	4	cis-regulatory regulatory element involved in the MeJA-responsiveness
circadian	ACGTG	1	cis-regulatory regulatory element involved in circadian control
RY-element	CATGCATG	1	cis-regulatory regulatory element involved in seed-specific regulation
CAAT-box	CAAT、CCAAT、CAAAT	38	common cis-regulatory element in promoter and enhancer regions
TATA-box	TATA、TATAA、TATAAA、ATTATAATATAT	43	core promoter element around -30 of transcription start
